# Brief Comment on the Article about Human Anaplasmosis by Arraga-Alvarado et al.

**DOI:** 10.4269/ajtmh.15-0379

**Published:** 2015-11-04

**Authors:** Irene Maury de Tamí, Irene Tamí-Maury

**Affiliations:** Instituto de Oncología y Hematología Ministry of Health Caracas, Venezuela Universidad Central de Venezuela Caracas, Venezuela E-mail: irenemaurydetami@yahoo.com.ve; The University of Texas MD Anderson Cancer Center Houston, TX E-mail: itami@mdanderson.org

Dear Sir:

We are writing to comment on the article by Arraga-Alvarado and others in the September 2014 issue of *The American Journal of Tropical Medicine and Hygiene*.^1^ To provide some necessary background information about the early recognition of ehrlichiosis and anaplasmosis in Venezuela, we would like to bring to the attention of the manuscript';s authors and readers an article published by de Tamí and others in 1994,^2^ in the third issue of the *Revista Científica de la Sociedad Venezolana de Bioanalistas Especialistas*, reporting the evaluation of 33 human blood samples and 50 canine blood samples from Caracas, Venezuela, for intra-platelet inclusions in buffy coat smears. In this pioneering study, platelet inclusion bodies (morulae) were identified in 32% of the canine blood samples and 45% of the human blood samples, suggesting that this microorganism could be implicated in the etiology of prolonged fever syndromes and idiopathic thrombocytopenias described in Venezuelan individuals. In 1996, a study conducted by Perez and others^3^ reported the isolation and antigenic and genetic characterization of a suspected new strain or subspecies of an *Ehrlichia*-like agent in monocytes from the blood sample of a Venezuelan veterinarian with assumed exposure to infected animals and ticks. Platelet inclusions were also reported in 14% of 87 Venezuelan patients infected with human immunodeficiency virus (HIV),^4^ suggesting the need for warning HIV-infected individuals and other at-risk populations about avoiding tick-prone areas, carefully examining animals (especially pets and livestock) with clinical signs of tick-borne diseases, and educating health-care providers in Venezuela on ehrlichiosis and anaplasmosis surveillance, prevention, and control.
